# Bioprocess performance analysis of novel methanol-independent promoters for recombinant protein production with *Pichia pastoris*

**DOI:** 10.1186/s12934-021-01564-9

**Published:** 2021-03-23

**Authors:** Javier Garrigós-Martínez, Kiira Vuoristo, Miguel Angel Nieto-Taype, Juha Tähtiharju, Jaana Uusitalo, Pauliina Tukiainen, Christian Schmid, Ilya Tolstorukov, Knut Madden, Merja Penttilä, José Luis Montesinos-Seguí, Francisco Valero, Anton Glieder, Xavier Garcia-Ortega

**Affiliations:** 1 Department of Chemical, Biological and Environmental Engineering, Universitat Autònoma de Barcelona, 08193 Bellaterra (Cerdanyola del Vallès), Spain; 2 Solutions for Natural Resources and Environment, VTT Technical Research Centre of Finland Ltd, Tietotie 2, Espoo, Finland; 3 Keck Graduate Institute, 535 Watson Dr, Claremont, CA USA; 4 Biogrammatics Inc, 2794 Loker Ave, West, Suite 103, Carlsbad, CA 92010 USA; 5 Institute of Molecular Biotechnology, Graz University of Technology, Petersgasse 14, 8010 Graz, Austria; 6 Bisy GmbH, Wuenschendorf 292, 8200 Hofstaetten/Raab, Austria

**Keywords:** *Komagataella phaffii* (*Pichia pastoris)*, Recombinant protein production, Methanol-free bioprocesses, Expression system characterisation, Promoter regulation, Bioprocess development

## Abstract

**Background:**

*Pichia pastoris* is a powerful and broadly used host for recombinant protein production (RPP), where past bioprocess performance has often been directed with the methanol regulated *AOX1* promoter (P_*AOX1*_), and the constitutive *GAP* promoter (P_*GAP*_). Since promoters play a crucial role in an expression system and the bioprocess efficiency, innovative alternatives are constantly developed and implemented. Here, a thorough comparative kinetic characterization of two expression systems based on the commercial PDF and UPP promoters (P_*PDF*_, P_*UPP*_) was first conducted in chemostat cultures. Most promising conditions were subsequently tested in fed-batch cultivations. These new alternatives were compared with the classical strong promoter P_*GAP*_, using the *Candida antarctica* lipase B (CalB) as model protein for expression system performance.

**Results:**

Both the P_*PDF*_ and P_*UPP*_-based expression systems outperformed similar P_*GAP*_-based expression in chemostat cultivations, reaching ninefold higher specific production rates (*q*_*p*_). *CALB* transcription levels were drastically higher when employing the novel expression systems. This higher expression was also correlated with a marked upregulation of unfolded protein response (UPR) related genes, likely from an increased protein burden in the endoplasmic reticulum (ER). Based on the chemostat results obtained, best culture strategies for both P_*PDF*_ and P_*UPP*_ expression systems were also successfully implemented in 15 L fed-batch cultivations where *q*_*p*_ and product to biomass yield (*Y*_P/X_*) values were similar than those obtained in chemostat cultivations.

**Conclusions:**

As an outcome of the macrokinetic characterization presented, the novel P_*PDF*_ and P_*UPP*_ were observed to offer much higher efficiency for CalB production than the widely used P_*GAP*_-based methanol-free alternative. Thus, both systems arise as highly productive alternatives for *P. pastoris*-based RPP bioprocesses. Furthermore, the different expression regulation patterns observed indicate the level of gene expression can be adjusted, or tuned, which is interesting when using *Pichia pastoris* as a cell factory for different products of interest.

**Supplementary Information:**

The online version contains supplementary material available at 10.1186/s12934-021-01564-9.

## Background

The non-conventional yeast *Komagataella phaffii,* widely known under the former name *Pichia pastoris*, is a distinguished host for recombinant protein production (RPP) [1–7] and metabolite production [8]. Among the many positive features that make *P. pastoris* a good choice for RPP, and historically one of the most relevant, is strong and tightly regulated expression based on the alcohol oxidase 1 promoter (P_*AOX1*_) [9–12]. When using the P_*AOX1*_ promoter, induction occurs in the presence of methanol, whereas glycerol or glucose fully repress expression [13]. De-repression is not sufficient for significant gene expression; therefore, a simple recombinant protein production process is typically divided into two phases. First, a glucose/glycerol-based batch phase, where a relatively high amount of biomass is generated without recombinant protein production. Subsequently, the methanol feeding phase triggers strong P_*AOX1*_-driven protein production. However, such tightly controlled induction and strong expression levels by P_*AOX1*_ causes operational drawbacks due to the use of methanol, including high oxygen requirements and heat production, as well as increased costs derived from methanol storage and handling [14, 15]. To address these challenges, mutated promoter variants [16] or co-substrate feeding strategies had been employed [17].

In order to open new opportunities, innovative alternatives are constantly developed, evaluated and implemented. In terms of promoter strength, the other methanol inducible promoters such as *DAS1* and *DAS2* (P_*DAS1;*_ P_*DAS2*_), demonstrate similar strength [18]. In addition to the numerous attempts that had been made to modify P_*AOX1*_ regulation by mutagenesis or synthetic fusions [19], the co-expression of transcription factors was demonstrated as an interesting alternative to induction by methanol. In addition, numerous methanol-independent expression systems have been developed and tested with promising results such as P_*GTH1*_, P_*CAT1*_, P_*THI11*_ P_*HpFMD*_ or P_*TEF*_, among others [20–25].

Historically, RPP improvements have been mainly obtained through strain and promoter system engineering [16, 26–29]. Multiple clones with different expression cassettes or random integration variants with the same expression cassette are tested and compared in parallel in shake flasks or microtiter plates. This approach is considered fast and cost-effective; however, most testing platforms do not allow control of key bioprocess parameters such as dissolved oxygen, pH, as well as growth and feed rates. Since these parameters affect target protein expression, selection of a “best performing” clone might not always be optimal. Accordingly, the performance of the production clones candidates should be compared in cultivation platforms such as bench-top bioreactors [22, 30, 31], and/or alternative systems that allow controlled substrate delivery. Using bioreactors, production processes can be carried out applying optimal ranges of the key bioprocess parameters [32, 33]. Chemostat systems, where cultures are maintained at non-dynamic, steady-state conditions, have become a valuable tool for bioprocess characterization and further optimization [34]. In this way, a full kinetic characterization of the candidate cell factories can be performed. Furthermore, interestingly, sampling for ‘omics’ analyses can be reliably carried out on cells from chemostat, where cultures have constant key process parameters, and the cell population is highly homogeneous [34].

Studies including precise strain characterization by chemostat cultivations, have revealed how the specific growth rate (*µ*) significantly affects recombinant protein production (RPP) rates [6, 15, 35–40]. Importantly, the relationship between *µ* and the specific production rate (*q*_*p*_), also called production kinetics, is dependent on both the expression system used and the recombinant protein expressed. In previous studies, García-Ortega et al*.* [37], and Nieto-Taype et al*.* [38] described a linear *µ*-*q*_*p*_ relationship when producing a human Fab, as well as the *Candida rugose* lipase 1 (Crl1), respectively, both under the control of the constitutive P_*GAP*_. The same trend was observed for the production of Lipase B from *Candida antarctica* using the constitutive *PGK* promoter [41]. These authors concluded that since the constitutive P_*GAP*_ has a pivotal role in the growth-associated glycolysis, therefore, one should expect the RPP to be growth-coupled. On the other hand, curved/non-linear *µ*-*q*_*p*_ trends were observed [21, 39, 42], suggesting non-coupled transcriptional regulation, or bottlenecks in the protein processing pathway. In particular, Garrigós-Martínez et al. [39] remarked that the *µ*-*q*_*p*_ bell-shaped trend observed in the P_*AOX1*_ regulated production of Crl1 was probably caused by an alternative transcriptional regulation. This conclusion was based on the determination that at different *µ,* target protein production profiles and the relative transcripts did not present the usual linearity of growth-coupled expression systems.

In this work, the performance characterization of two novel expression systems for RPP with *P. pastoris* are based on: (1) The new *PDF* promoter (P_*PDF*_, a commercial variant of the *Hansenula polymorpha FMD* promoter [25, 43], which drives strong transcription by simple methanol-free de-repression and can be also further induced with methanol), and (2) *UPP* promoter (P_*UPP*_, a constitutive commercial variant of a *Pichia* promoter called *GCW14*, [23]). Both promoter systems have been thoroughly studied and compared with P_*GAP*_*,* the most well characterized constitutive promoter, considered a reference standard for methanol-free expression systems. Expression strains for the lipase B from *Candida antarctica* (CalB) were constructed, all with the same parental strain, gene dosage and identical vectors, except for the promoter sequence driving *CALB* gene expression. To compare these expression systems, a set of chemostat cultivations designed to assess the effect of different *µ* values on the production kinetics was performed. Furthermore, *CALB* transcript levels were determined and compared to the expression levels for each condition tested in chemostat. Finally, working at the *µ* ranges that generated the best results in chemostat mode, the same selected clones were cultivated in 15 L fed-batch processes to evaluate their performance in this operational mode.

## Results and discussion

### Strain generation, screening and gene dosage

Isogenic clones were generated to compare the performance of the promoters P_*UPP*_, P_*PDF*_ and P_*GAP*_ for the CalB expression as a model recombinant protein. Considering the potential clonal variability often observed in *Pichia* clone generation methods, care was taken to select a clone for each expression system with a single expression cassette integrated into the genome [29, 44]. Subsequently, around 90 individual transformants were analyzed in a high-throughput screen based on deep well plate (DWP) system, to develop a “landscape” of expression data for clone characterization according to Weis et al*.* [45]*.* Putative single-copy integration transformants for each of the different promoter constructs were picked from the majority of transformants which showed very similar lipase activity in the supernatant after cultivation and induction in 96-DWP and an initial screen measuring CalB activity of secreted reporter enzyme. Among the discarded clones, secreting higher amounts of CalB were suspected to be associated with multicopy or random integration events; while lower activity observed in some clones might be from with detrimental effects exerted by ectopic integration [46]. Subsequently, a second round of DWP screening among the potential single copy integration candidates was performed, which were tested at least the biological triplicates (data presented on Additional file 1: Figure S1A, B, C). Therefore, for the candidate clones, gene dosage was determined by droplet digital PCR (ddPCR, data presented on Additional file 2: Tables S2A, B) to confirm each construct only contained a single-copy of the respective expression vector in the *Pichia* genome. Confirmed single copy clones for each expression system presenting an average CalB production were therefore selected to start the expression systems characterization, thus ensuring that production differences are only a result of the effect of each promoter’s specific influence on CalB recombinant expression, and not due to a different gene dosage.

### Physiological state comparison of the *P. pastoris* clones harboring different expression system

Chemostat cultivations were performed with one selected CalB production clone for each of the three different expression systems (GAP-C, PDF-C and UPP-C). This comparison test was performed at three different dilution rates (*D*): 0.05 h^−1^, 0.10 h^−1^ and 0.15 h^−1^. This characterization allowed to determine the range of dilution rate to significantly improve CalB production in subsequent fed-batch (FB) cultivations.

**Fig. 1:**
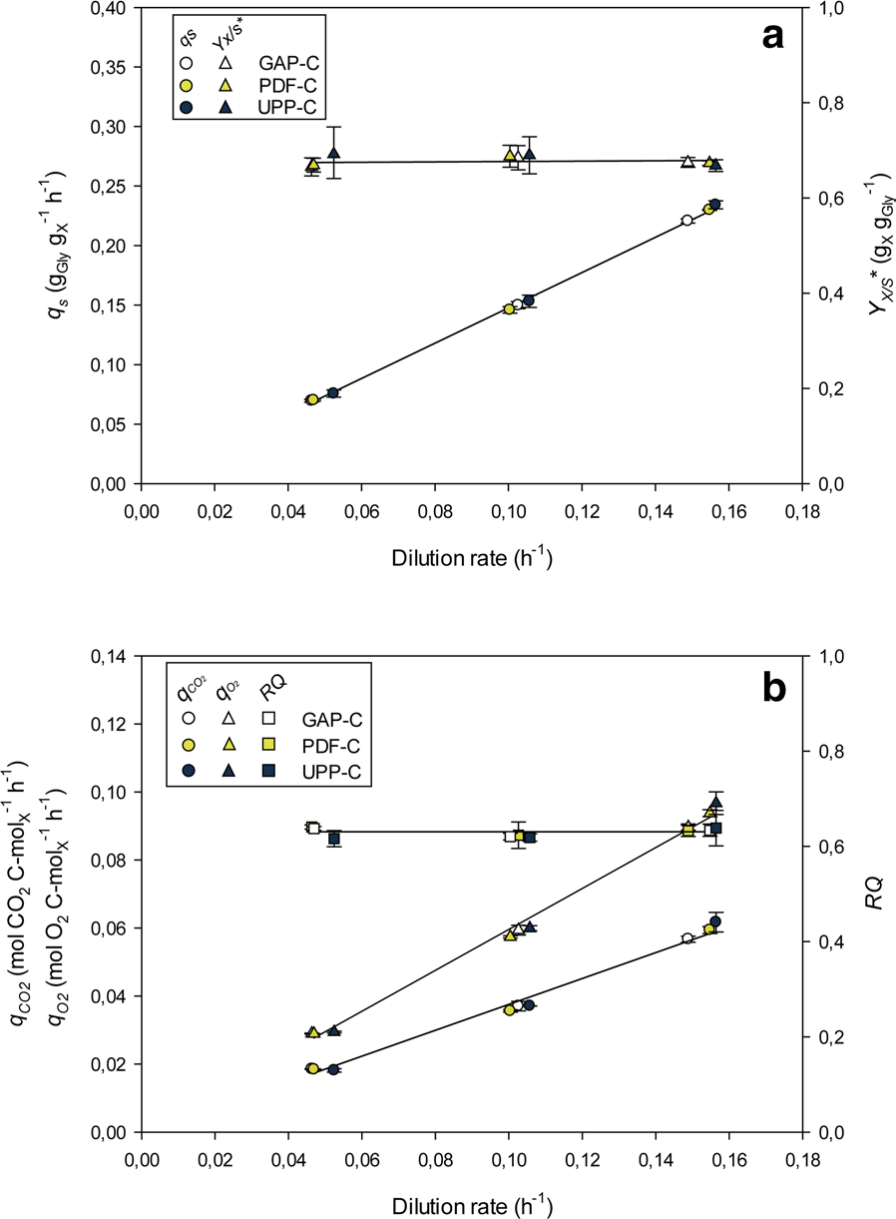
Physiological state indicators of *Pichia pastoris* CalB producer clones-GAP-C, PDF-C, UPP-C—in chemostat cultivations. **a** Specific glycerol consumption rate (*q*_*s*_), overall glycerol-to-biomass yield (*Y*_*X*/S_*). **b** Specific oxygen uptake rate (*q*_O2_), specific carbon dioxide production rate (*q*_CO2_) and respiratory quotient (RQ). Error bars represent the standard deviation of two biological replicates

P_*UPP*_ and P_*PDF*_ clones had significantly higher expression levels than those based on P_*GAP*_. Furthermore, high levels of recombinant protein expression have been shown to cause a burden on the protein secretion machinery likely due to an overload of the processing capacity [47, 48]. Therefore, an impact of the three expression systems on the physiological state was tested in chemostat cultivations by analyzing glycerol and O_2_ consumption rates, and CO_2_ production rates (Fig. 1).

In this regard, no significant differences were evident across the *D* tested. The specific glycerol consumption rate (*q*_*s*_) and overall biomass-to-substrate yield (*Y*_X/S_*) were rather similar. As expected, *q*_*s*_ increases linearly over the *D*, whereas *Y*_X/S_* values were constant (only slight differences could be observed at the highest D). All the clones presented similar specific CO_2_ production rates (*q*_*CO2*_) and specific O_2_ consumption rates (*q*_*O2*_) and followed standard linear trends. Consequently, similar respiratory quotient values were exhibited by all the clones studied (RQ, i.e. about 0.62, see Fig. 1b). Based on the analysis at macrokinetic level, it can be stated that the higher CalB production provided by the new generation expression systems based on the promoters P_*PDF*_ or P_*UPP*_ did not alter any of the studied physiological parameters compared to the GAP-C, which presents lower *CALB* expression levels.

### Novel expression systems outperform P_*GAP*_-based CalB production

Compared to P_*GAP*_, both of the new promoters resulted in notably higher *q*_*p*_ values, between 4 and ninefold higher at any *D* (Table 1). UPP-C also had *q*_*p*_ values significantly higher than PDF-C at the lowest and middle *D*. At the highest *D*, UPP-C was similar to PDF-C, with only slightly higher *q*_*p*_.

**Table 1:** Comparison of the main production parameters obtained in chemostat and fed-batch cultures with the producer clones at different specific growth rates (*µ*)

Clone	Operational mode	*Nominal µ*	*Experimental µ*	*q*_*p*_	*Y*_P/X_*
		h^−1^	h^−1^	AU g_x_^−1^ h^−1^	AU g_x_^−1^
GAP-C	Chemostat	0.050	0.046	1.16	24.9
		0.100	0.103	2.24	21.9
		0.150	0.149	2.74	18.4
PDF-C	Chemostat	0.050	0.047	7.20	153
		0.100	0.100	10.8	108
		0.150	0.155	10.8	70.1
	Fed-batch	0.050	0.042	9.20	219
		0.100	0.087	13.1	150
UPP-C	Chemostat	0.050	0.052	10.3	197
		0.100	0.106	12.9	122
		0.150	0.156	11.4	72.7
	Fed-batch	0.050	0.051	11.1	217
		0.100	0.084	9.95	102

**Fig. 2:**
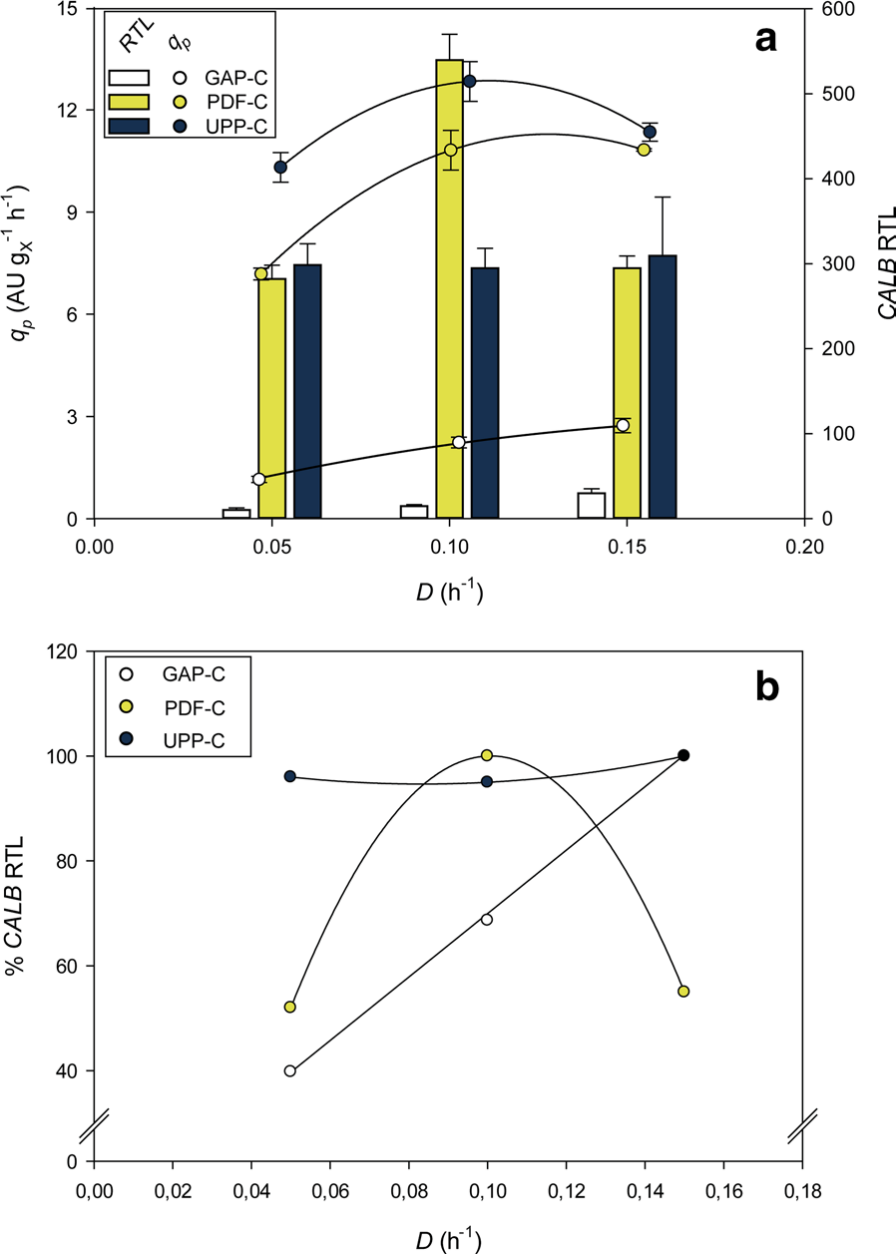
Comparison of the overall CalB product-to-biomass yield (*Y*_*P*/X_*) in chemostat cultivations for the three expression systems tested. Error bars represent the standard deviation of two biological replicates

Different production kinetic profiles, *q*_*p*_ at different *D*, were obtained for all three expression systems compared (Fig. 2a). UPP-C presented a bell-shape profile with a maximum at mid *D*, 0.10 h^−1^. On the other hand, PDF-C generated a clearly saturated profile. And, GAP-C had a relatively linear pattern, with a slight saturation trend at higher *D* values. This result differs from other examples reported using P_*GAP*_ in which *q*_*p*_ clearly increases linearly with *D* [37, 38]. Thus, these results indicate that production kinetics, in most cases, are protein dependent.

**Fig. 3:**
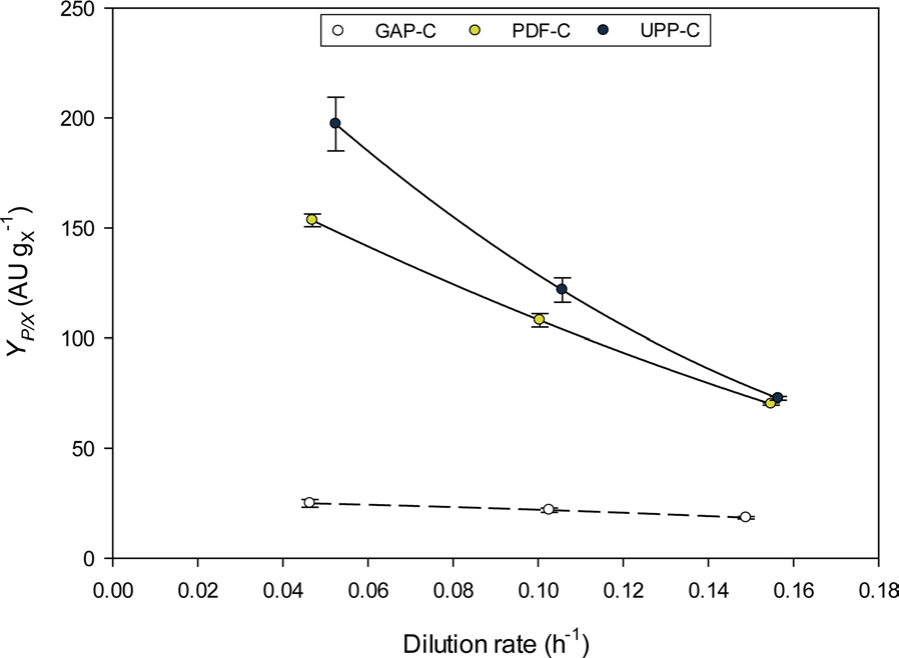
**a** CalB production kinetics (*q*_*P*_ vs *D*) and *CALB* relative transcription levels determined in chemostat cultivations for the three expression systems studied. Transcript levels were normalized to the levels of the *MTH1* transcript, which was used as housekeeping gene for the analysis. Error bars represent the standard deviation of two biological replicates. **b** Percentage of *CALB* relative transcription levels respect to the maximum observed for the corresponding expression system

Another important key parameter to be considered is the overall product to biomass yield (*Y*_P/X_*); it determines the overall capacity of cells to produce recombinant protein under certain conditions. The P_*UPP*_ and P_*PDF*_ expression systems are similar to other systems where increasing *D* is detrimental for Y_P/X_* [38, 39], as shown in Fig. 3. As observed with *q*_*p*_, the biggest difference between UPP-C and PDF-C *Y*_P/X_* values was demonstrated at the lowest *D*, yet similar at higher *D*. Importantly, the highest *Y*_P/X_* values for UPP-C and PDF-C were notably higher than those obtained with the GAP-C (i.e. up to 8.9-fold higher with UPP-C at 0.05 h^−1^) (Fig. 3).

Based on CalB production-related parameters *q*_*p*_ and *Y*_P/X_*, both novel expression systems should be considered good candidate promoters to produce recombinant proteins. Different conditions could be optimal depending on the objective: to reach higher product titer, or maximal productivity. If the objective is the highest protein titer, the lowest *D* should be selected [38, 39, 49], especially in the case of UPP-C, where *Y*_P/X_* reductions with *D* were more pronounced than with PDF-C. On the other hand, in order to maximize *q*_*p*_, the conditions recommended for high production should be at a *D* of 0.10 h^−1^, observed most markedly with PDF-C, where *D* has a bigger impact (Fig. 2a).

### The new promoters enable increased tunability of recombinant protein expression processes in *Pichia pastoris*

mRNA levels are not always directly correlated with the level of recombinant protein production obtained [22, 38, 39], especially since high *CALB* transcript levels can cause enhanced cellular stress [50]. In the present work, variable *CALB* transcript levels were observed in all three expression systems compared: GAP-C, UPP-C, and PDF-C. As shown in Fig. 2b, a linear profile of *CALB* relative transcript levels (RTL) was observed across *D* for GAP-C, confirming the widely reported constitutive and growth-coupled regulation pattern with P_*GAP*_. For this GAP-C, even both RTL-*D* and *q*_*p*_-*D* present similar profiles, a slight saturation trend of *q*_*p*_ can be observed at high *D* (Fig. 2a), likely because the low production rates observed with P_*GAP*_ are not likely to overload of the processing and secretory capacity. For UPP-C, only slight differences in *CALB* RTL were observed among the different *D* tested. Therefore, the regulation of *CALB* expression under P_*UPP*_ control should be considered growth independent. Strikingly, the *CALB* mRNA expression patterns do not correlate with the bell-shaped *q*_*p*_ profile described in the previous section (highest at a 0.10 *D*, Fig. 2a). For the PDF-C, RTL presents a bell-shape trend, while the *q*_*p*_-*D* profile presents a saturation pattern. Therefore, according to the RTL results (Fig. 2b), the P_*PDF*_-based expression system exhibits a growth-rate dependent regulation, which thus can be considered a system with a promising tunable expression pattern.

**Fig. 4:**
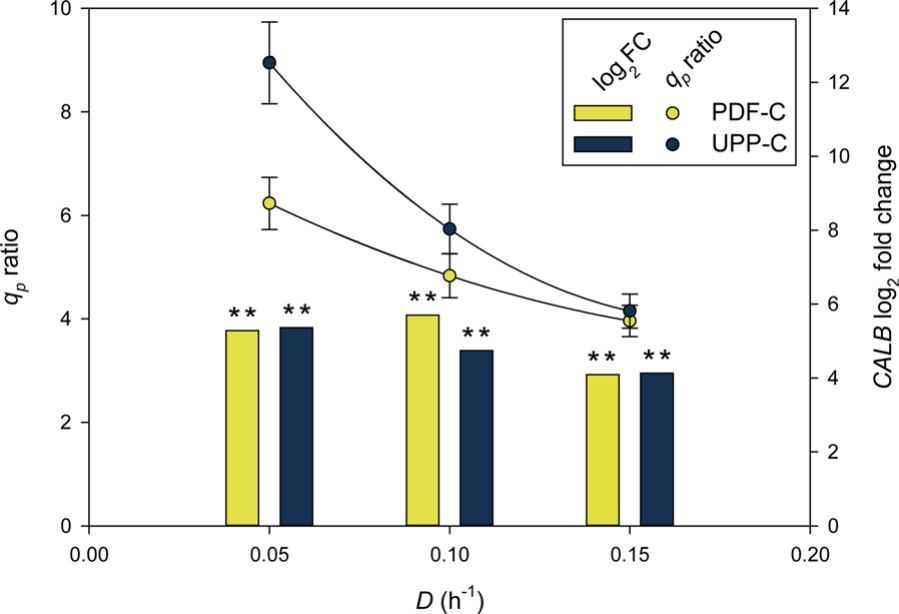
Effect of dilution rate on the *q*_*p*_ ratio and the *CALB* differential RTL calculated as log_2_ fold change relative to GAP-C values. GAP-C values were used as a control for ratio calculations. P-values (t-test) were calculated in order to determine the *CALB* expression significance between producer clones (* significance level P ≤ 0.05; ** significance level P ≤ 0.01)

As presented in Fig. 4, the comparison of *CALB* expression regulated by P_*UPP*_ and P_*PDF*_, relative to the P_*GAP*_, illustrates an interesting contrasting behavior. The weaker, growth-coupled, P_*GAP*_-based expression system performs better at higher *µ;* demonstrated here with the single-copy expression strain. In this case, the highest target transcription levels may result in a recombinant protein “burden” can still be sorted, or processed, properly in the ER. On the other hand, with both novel promoters, *CALB* transcription levels and specially *q*_*p*_ ratios, decrease over *D*, thus indicating that high specific growth rates are detrimental for these more productive systems. Both UPP-C and PDF-C generated *CALB* transcripts, as well as secreted protein at significantly higher levels than GAP-C. The higher transcript levels may be overwhelming the secretory pathway, triggering the unfolded protein response (UPR). Therefore, UPP-C and PDF-C might be better at low and medium *µ*, when most of transcription can generate protein, as is demonstrated by the higher productivity rates. Consequently, suitable conditions to balance both growth and protein production are needed to improve production protocols.

### UPR influence on CalB production

**Fig. 5:**
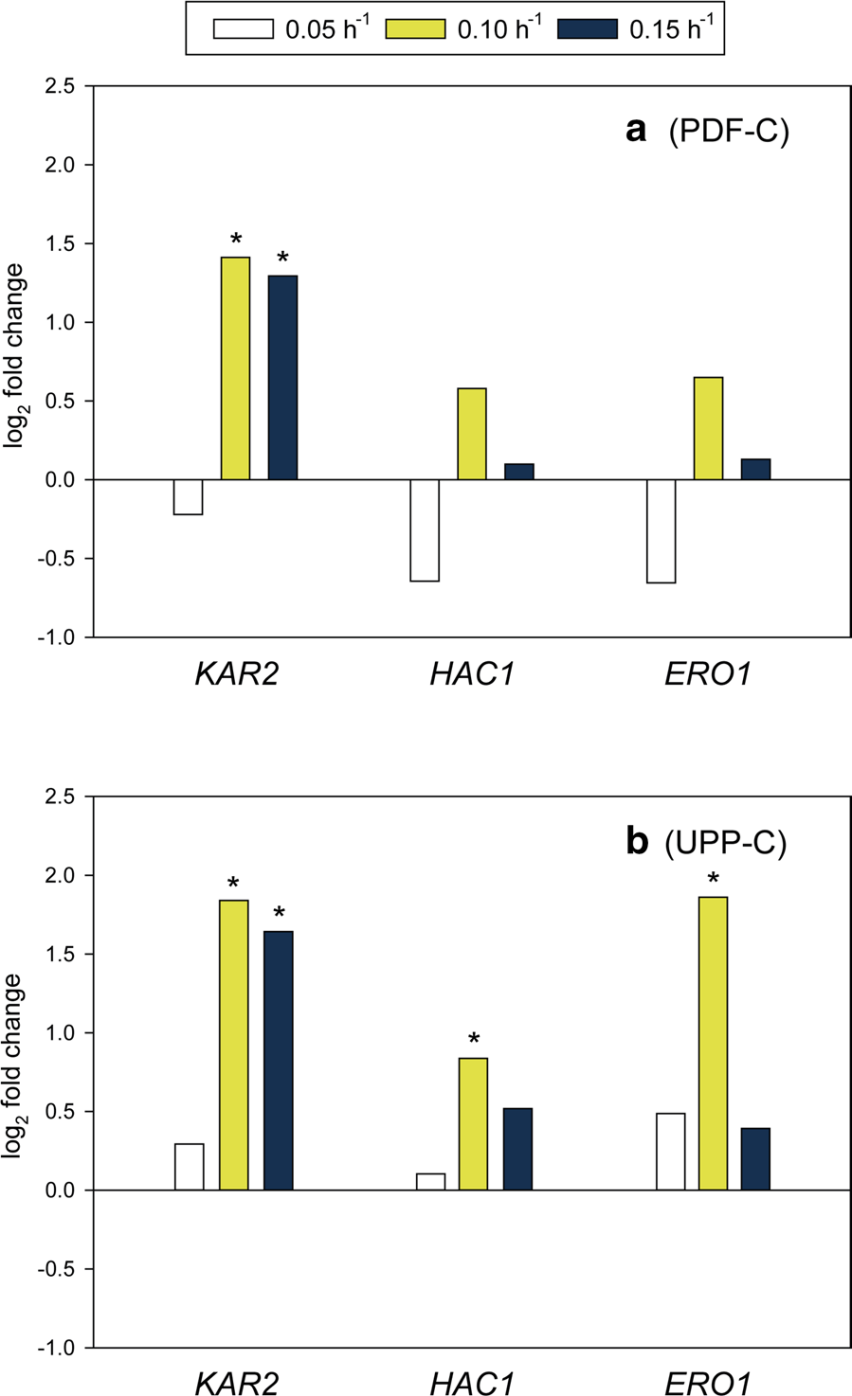
Transcriptional levels of three different UPR-related genes at the different dilution rates. **a** PDF-C, and **b** UPP-C (presented as a comparison of transcript levels with the levels from the control GAP-C, log_2_ scale). P-values (t-test) were calculated for all the genes and conditions in order to determine the gene expression significance between producer clones (* significance level P ≤ 0.05)

In order to assess potential endoplasmic reticulum (ER) stress derived from the excessive heterologous protein production, the expression of key UPR genes were analyzed. The reporters selected were two well-known ER-resident chaperones, *KAR2* and *ERO1,* and a gene product generally considered to be an UPR master regulator, *HAC1* [2, 51, 52]. The relative transcription levels of these three UPR related genes were measured in UPP-C and PDF-C and compared to the GAP-C. Data is presented as a log_2_ fold change relative to the GAP-C levels (Fig. 5).

Overall, the expression of all three of the UPR reporter genes, *HAC1*, *KAR2* and *ERO1,* had similar expression patterns across all the *D* rates tested. Their expression was the highest when the clones were cultivated at intermediate *D* of 0.10 h^−1^, whereas only moderate expression increases were found at *D* 0.15 h^−1^. These results are in-line with other published work reporting that not only recombinant protein production, but also *µ,* having a significant impact on UPR induction [36, 53]. The cited studies are also similar to the data presented here, showing unremarkable UPR levels at lower *µ,* whereas *µ* increases led to UPR upregulation.

Strikingly, the growth-coupled expression of *CALB* by GAP-C was the only case in which the *CALB* RTLs presented a similar pattern to the *q*_*p*_. The UPP-C *q*_*p*_ values mimic the UPR sensor gene expression profiles across the *D* tested, regardless of *CALB* transcription rates, which were rather similar for all the *D* rates. Therefore, UPR might have an influence in subsequent steps of CalB processing and secretion. The UPR impact on *CALB* expression is demonstrated by comparing *q*_*p*_ and *CALB* RTL values at *D* 0.10 h^−1^. In the Fig. 5, UPR-associated gene expression is higher for UPP-C (Fig. 5b) than PDF-C (Fig. 5a) and may explain why UPP-C *q*_*p*_ is higher than PDF-C *q*_*p*_ at this *D* despite UPP-C presenting 84% less *CALB* RTL. In this sense, it has been also described that the co-expression of protein disulfide isomerase, which is also upregulated at higher UPR, enhances active lipase production by *P. pastoris* [54]. Lastly, the comparison between two *D* conditions for PDF-C in continuous cultivations supports this hypothesis. At both *D* 0.05 h^−1^ and *D* 0.15 h^−1^, the *CALB* RTL levels are rather similar. However, the UPR-related gene expression is growth coupled, enhanced at higher *D*. The increased UPR could be contributing the 50% higher *q*_*p*_ observed at the highest *D,* even though the target gene RTL levels are rather similar.

Together, these analyses indicate that *q*_*p*_ for CalB in *P. pastoris* is influenced by several factors: heterologous gene transcription rates, recombinant protein-associated UPR, and *D*-associated UPR. Comparing the different alternatives for methanol-free expression presented in this work, it could be observed that the new generation constructs, based on the *PDF* and *UPP* promoters, allowed to achieve *CALB* transcription levels of up to eightfold higher than with P_*GAP*_-regulated expression, for all the *D* tested. However, at the high target protein expression levels, a direct correlation between *CALB* RTL and *q*_*p*_ was not observed. In contrast, higher *q*_*p*_ values were usually observed at *D* conditions with enhanced expression of UPR-related genes, suggesting a relevant impact of UPR on CalB production.

### Fed-batch cultures for further scalable bioprocess development

**Fig. 6:**
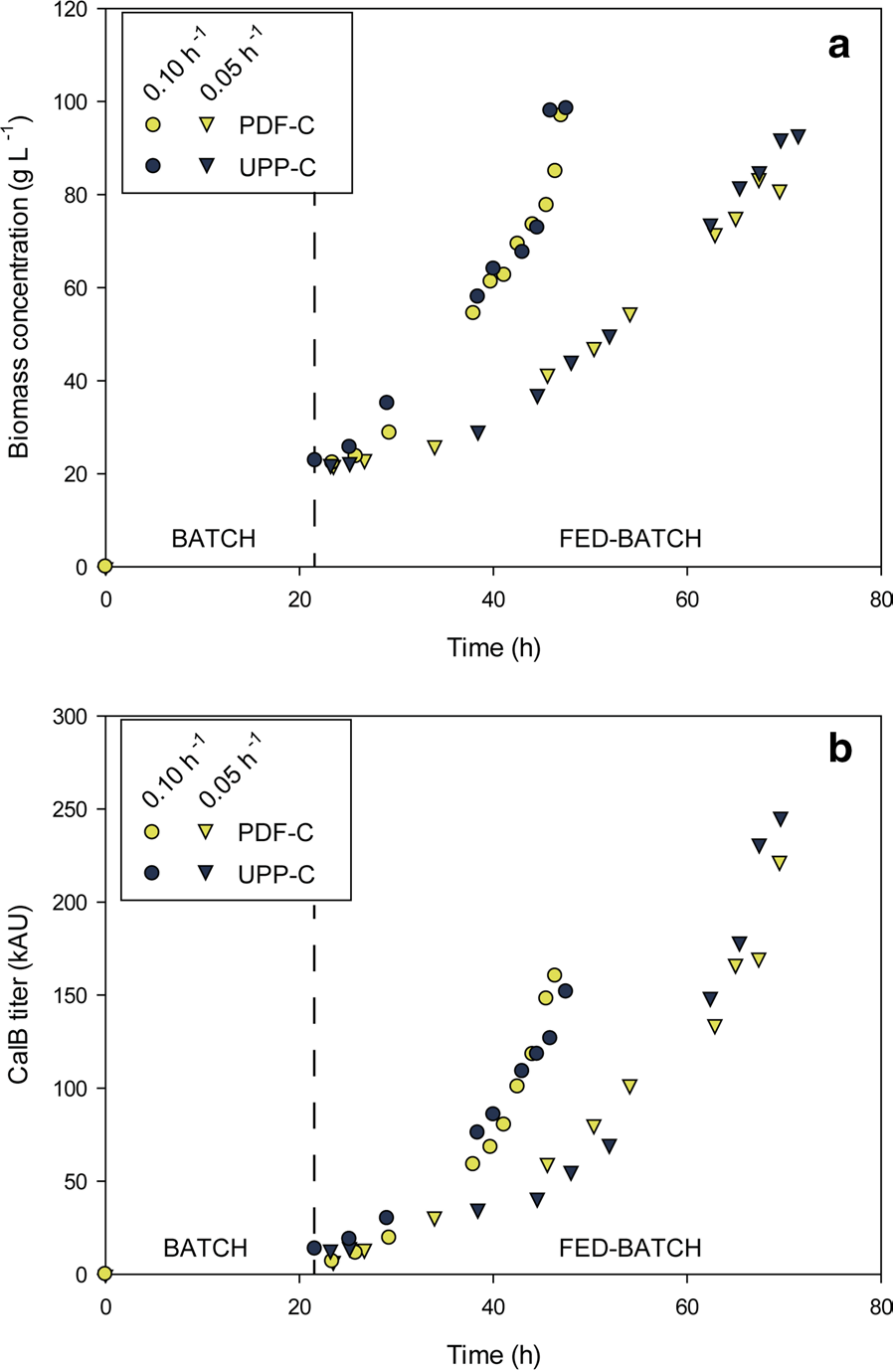
Fed-batch culture time profiles of Biomass (**a)** and CalB production (**b**) expressed as cell concentration (g L^−1^) of dry cell weight concentration (DCW) and total activity units (kAU), respectively. Vertical lines separate batch and fed-batch phases

Through chemostat cultivations, a systematic characterization of the three expression systems studied at different dilution rates was carried out, generating information both at macrokinetic, stoichiometric and transcriptional level. From these results, the range of *µ* that improves CalB production were determined in order to achieve higher product yields and/or productivities. Ideally, the best *µ* values found in chemostat cultivations should be implemented to fed batch cultures (FB), which is currently the most widely used scalable operational mode for industrial production of recombinant proteins. However, production kinetics may present relevant differences between the different operational modes [38, 39]. Therefore, fed-batch cultivations were also conducted to confirm CalB production kinetics pattern determined in chemostat cultivations. Carbon-limited fed-batch cultivations, the culture strategy usually considered as most efficient with *P. pastoris* methanol free processes*,* were performed with the UPP-C and PDF-C to obtain biomass and CalB production profiles (Fig. 6). Accordingly, based on the results obtained in chemostat cultures in which strong expression systems perform better at mid-low *µ*, the *µ* of 0.15 h^−1^ was discarded for further fed-batch implementation.

As expected, biomass production of all the cultures presented the targeted exponential profile, reaching a maximum between 90 and 100 g L^−1^ of dry cell weight (DCW), which is considered a standard endpoint for *Pichia* high-cell density fed-batch (Fig. 6a). CalB production, expressed in activity units (kAU), also increased pseudo-exponentially over time (Fig. 6b). Product titers obtained at the lowest *µ* tested (~ 0.05 h^−1^) were substantially higher than with the intermediate *µ* (~ 0.10 h^−1^), being 38% higher with PDF-C and 67% with UPP-C. Therefore, *Y*_P/X_* values were also markedly higher at the low *µ* cultures (Table 1). Comparing product-related parameters between chemostat and FB cultivations, PDF-C performed better in FB mode (24% higher, on average *q*_*p*_, and 41% on average higher *Y*_P/X_* at the equivalent *µ*, Table 1). On the other hand, UPP-C presented smaller differences of *q*_*p*_ and *Y*_P/X_* values at low *µ*, on average below 10%. Strikingly, for the range *µ* 0.10 h^−1^*,* UPP-C presented performance parameters were significantly worse in FB cultures relative to the chemostat cultures (Table 1). With respect to product quality and purity, it is worth to mention that the supernatant impurity fingerprints were similar between all four fed-batch cultivations; all had very low levels of *Pichia* native proteins secreted to the culture broth. Additionally, as observed in the SDS-PAGE presented, no signs of CalB degradation is observed, indicating protein quality is similar between the different expression systems and fed-batch cultivations (Additional file 3: Figure S3).

At the end of the batch phase, a twofold higher CalB titer was obtained with UPP-C compared to PDF-C. This points to a difference in the expression systems regulation in presence of excess carbon, a situation that only occurs during the batch phase. Whereas, P_*UPP*_ presents a constitutive and strong expression in excess of C-source, P_*PDF*_-regulated expression was repressed and increase under C-limiting conditions, such as at the end of the batch phase, and during the fed-batch phase. Thus, P_*PDF*_ generates an interesting tunable expression system, allowing simple uncoupling of biomass growth and achieving different transcription levels without altering the carbon source.

## Conclusions

In this study focused on developing methanol-free alternatives for RPP with *P. pastoris*, a macrokinetic characterization of two promising expression systems were conducted in chemostat cultures, P_*UPP*_ and P_*PDF*_, benchmarking their performance with P_*GAP.*_ In terms of substrate and respiration-related parameters, all three expression systems behaved similarly, suggesting that the potential differences in CalB production does not significantly alter the yeast homeostasis in chemostat cultivations. Overall, the CalB production kinetics with the two novel expression systems generated significantly higher levels of recombinant protein than the reference, GAP-C, up to ninefold higher in terms of *q*_*p*_. The differences in product-related parameters were primarily attributed to the significantly higher *CALB* transcription levels. Interestingly, under carbon-limiting conditions, the P_*PDF*_-based expression system showed a *D*-dependent tunable expression, while P_*UPP*_-regulated expression was more constant, independent of the growth rates tested. Furthermore, an UPR up-regulation was noted most markedly with the UPP-C at *D* = 0.1 h^−1^. At this dilution rate, the three UPR reporter genes monitored were at their highest level. Notable is that the highest *q*_*p*_ was also at this *D* condition.

The chemostat results were used to design strategies based on the *µ* ranges that provide the best production results for its further implementation in fed-batch cultivations. Thus, both novel expression systems, based on P_*UPP*_, P_*PDF*_*,* were also tested in this operational mode. The difference in regulation patterns was reproduced in a fed-batch mode as UPP-C had around twofold higher CalB production than PDF-C at the end of batch phase, illustrating a strong constitutive *CALB* expression under P_*UPP*_ regulation. On the other hand, highest *CALB* expression in the PDF-C was obtained under C-limiting conditions, in which the expression is derepressed, thus presenting an interesting tunable expression pattern. Concerning the production kinetics, UPP-C showed much better performance at low *µ* in fed-batch, as the *q*_*p*_ at this *µ* outperformed the levels obtained at mid *µ* by 11%. PDF-C expression, on the other hand, was enhanced at mid *µ,* as *q*_*p*_ was significantly higher than under low *µ* conditions. In all cases, no significant difference in the product quality was observed among the different fed-batch cultivations performed, presenting for all cases low levels of protein impurity fingerprints and no signs of CalB degradation.

This work, testing alternative promoter designs, validate the approach that confirms the transferability from small scale screenings to its characterization in chemostats. Most importantly, outcomes obtained during the characterization should be considered as highly relevant to establish further strategies to be finally industrially implemented at large scale. Furthermore, these production conditions should also be taken into consideration for further improvements of the host at a molecular level, closing the duty cycle of synthetic biology based on iterative Design-Build-Test-Learn steps (DBTL). The successful results obtained in this work, are expected to make this approach transferable to other processes based on other expression systems and also different microbial cell factories towards rationally improve the efficiency of bioprocesses.

## Materials and methods

### Clone construction, selection and expression testing

The parental strain, *P. pastoris* BSYBG11(*aox1-/Mut*^*S*^), which is a BioGrammatics (Carlsbad, CA) *Pichia pastoris* BG11 strain, deposited at Bisy in Austria, was transformed with each of the expression vectors, only differing in the respective promoters. This strain is derived from the BioGrammatics *K. phaffii* (*P. pastoris)* strain BG10 but with a slower methanol metabolization phenotype, *Mut*^*S*^ [55]. The transformation method to express *Candida antarctica* lipase B (CalB) under the regulation of P_*GAP*_, P_*UPP*_ and P_*PDF*_ has been described elsewhere [56]. Recombinant vectors with the selected promoters were based on pPpT4_Alpha_S vector [57]. To avoid biasing of the results by transformant variability, low amounts of the linearized plasmid DNA (< 1 µg of DNA) were used to avoid multi copy expression cassette integration as described elsewhere [29, 44].

Candidate clones expression screening was done at microscale cultures in deep well plates (DWP´s), as described by Krainer et al. [58] with minor modifications. During the induction phase, methanol from BMM2 and BMM10 was replaced with glycerol as the carbon source with 1% and 5% glycerol (w/v), respectively. All media were buffered at pH 7.0.

An initial screen in DWP’s allowed to select the producer candidates from the clonal variability after transformation. Furthermore, a second screening employing DWP’s with seven replicates per clone was used to validate previous results with biological replicates towards the selection of producer clones that integrated only one copy of the desired expression cassettes. These final clones were used in the chemostat and fed-batch cultivations.

### Gene dosage determination

The gene dosage/copy number was determined for each selected producer clone using droplet digital PCR (ddPCR) as described elsewhere [38, 39]. ddPCR was performed with primers to amplify the *CALB* gene present in the expression cassette, as well as with primers for amplification of the Actin 1 gene (*ACT1*) as a reference. The *ACT1* gene had been demonstrated before, and thus to be a reference for single copy gene of the haploid *P. pastoris*. The list of primers used is presented as Additional file 2: Table S2A.

### Total RNA extraction, cDNA synthesis and transcript level determination

RNA was isolated from 1 mL culture samples taken from the chemostat culture broth as defined by Landes et al. [22]; RNA was prepared using the SV Total RNA Isolation System (Promega, Madison, Wisconsin, US) following the manufacturer’s instructions.

RNA integrity was checked by agarose electrophoresis and RNA concentration was measured with Nanodrop 2000 (Thermo Scientifc™, Waltham, MA, US).

cDNA was synthetized using iScript™ cDNA Synthesis kit (Bio-Rad, Hercules, CA, USA), following the manufacturer’s instructions. Primers were designed to analyze the relative transcript levels (RTL) of the following target genes by qPCR: *CALB*, *KAR2* and *ERO1* (two ER-resident chaperones), and *HAC* (UPR master regulator). *MTH1* gene was used as housekeeping gene for the transcriptional analysis. The qPCR procedure, including equipment, qPCR master mix solution and housekeeping gene is detailed in prior work [39]. The annealing-extension temperature was adjusted to 59 °C. The list of primers used is presented as Additional file 2: Table S2A.

### Chemostat cultivations

Chemostat cultivations were performed in duplicate, all in 2 L Biostat B plus Bioreactors (Sartorius Stedim, Goettingen, Germany) according to García Ortega et al. [37]. Batch and chemostat media compositions are stoichiometrically identical to the detailed in the reference [37]; however, the concentrations were reduced by half.

Cultivation conditions were monitored and controlled at the following set points: pH, 5.0 with addition of 15% (v/v) ammonium hydroxide; temperature, 25 °C; stirring rate, 700 rpm; air flow, 0.8 vvm and pO_2_ values were variable depending on the dilution rate. pO_2_ values were above 20% in all conditions tested. An exhaust gas condenser with cooling water at 4 °C minimized mass loses by water evaporation and other possible volatile compounds.

A broad range of dilution rates were covered for the three expression systems tested. Taking into consideration that 0.19 h^−1^ was the *P. pastoris µ*_*max*_ of GAP-C at 25 °C (data not shown), the following dilution rates were used: 0.05 h^−1^, 0.10 h^−1^ and 0.15 h^−1^ (dilution rates were tested as low, middle and high growth rate conditions, respectively). In order to ensure that the steady state was reached, the stability of the parameters of interest were monitored from the third residence time until the fifth one, where samples were taken.

### Fed-batch cultivations

Fed-batch cultivations were performed in duplicate, all in New Brunswick BioFlo 510 bioreactor (Eppendorf, Germany), connected to BioCommand Control software. Batch media composition was the same as that used in the chemostat runs, stoichiometrically identical to that used in prior work [49], except glycerol, instead of glucose, was used in the fed-batch feeding. The cultures were grown at 25 °C under overpressure (0.2 bar) and had a 7.5 L starting volume, including 1 L seed. The pH was kept at 5.0 by the automated addition of 12.5% NH_4_OH. Dissolved oxygen (DO) was maintained above 30% of air saturation with the automatic control of stirrer speed (400–700 rpm), constant airflow 10 L min^−1^, and enriched with O_2_ when needed (0–50% of total inlet flow rate).

Glycerol feeding was started upon depletion of batch medium glycerol. An exponential pre-programming feeding rate was performed to maintain the specific growth rate (0.05 h^−1^ or 0.10 h^−1^) constant at the selected set-point. All cultivations were grown under carbon-limiting conditions. The procedure is described in detail elsewhere [49].

### Biomass determination

Biomass concentrations were measured in triplicate as DCW values, as described elsewhere [59]. The relative standard deviation (RSD) of the measurements was about 4%.

### Quantification of the carbon source and byproducts

The concentration of both glycerol and the potential fermentation byproducts, were measured by HPLC. The column and the program used are described elsewhere [60]. RSD was invariably less than 1%.

### Off-gas analyses

A BlueInOne Cell gas analyzer (BlueSens, Herten, Germany) was used for monitoring the CO_2_ and O_2_ molar fraction of the chemostat cultivations off-gas. Off-gas pressure and humidity measurements were used to calculate the oxygen uptake rate (OUR), carbon dioxide evolution rate (CER), and their corresponding specific rates (q_O2_ and q_CO2_) and respiratory quotient (RQ), as previously described. RSD was less than 5% in all cases.

### Enzymatic analyses

CalB activity was determined by an esterase activity assay, similar to the assay described by Krainer et al. [61]. Briefly, 100 μL of culture supernatant was mixed with 900 μL of fresh assay solution containing 4 mM p-NPB in 300 mM Tris–HCl, pH 7.4, 1% acetone. The absorbance increase at 405 nm was monitored at 30 °C for 2 min (Specord 200 Plus spectrophotometer from Analytic Jena Germany). One activity unit was defined as the amount of enzyme needed to release 1 μmol of p-nitrophenol per minute under assay conditions. RSD was less than 4%.

SDS-PAGE analysis were performed with the culture supernatants collected during the bioreactor cultivations, which were diluted into water prior to loading into precast 4–20% kD Criterion TGX Stain-Free Gel (Biorad, Hercules, CA, USA). Fifteen µL of the diluted samples were mixed with 5 µL of 4 × loading buffer (20% glycerol, 4% SDS, 0.3 mM bromophenol blue (Merck), 10% β-mercaptoethanol, 0.1 M Tris, pH 6.8), and incubated at 95 °C for 5 min. Samples were cooled, centrifuged quickly, and 19 µL loaded into 4–20% kD Criterion TGX Stain-Free Gel together with Precision Plus Protein molecular weight marker (Biorad, Hercules, CA, USA). The visualization of the gel was performed using the Gel Doc EZ (Biorad, Hercules, CA, USA).

### Process parameter determination, consistency test and data reconciliation

#### Mass balance and stoichiometric equations

All equations used to calculate yields and rates are based on mass balances at continuous and fed-batch operation and can be found elsewhere [11, 37]. The *P. pastoris* elemental composition grown on glycerol as the sole C-source was determined as previously reported [62]. Carbon and electron balances were checked and less than 5% of deviation was observed prior to reconciliation.

#### Consistency test and data reconciliation

Measurement consistency was checked by using the standard test with carbon and electron balances as constraints. Both online and offline measurements enabled the calculation of five key specific rates in the black-box process model: biomass generation (*μ*), glucose uptake rate (*q*_*s*_), product generation rate (*q*_*p*_), oxygen uptake rate (*q*_*O2*_) and carbon dioxide production rate (*q*_*CO2*_). The method used for this purpose is described in detail elsewhere [11].

## Supplementary Information

**Additional file 1: Table** **S1.** Figures that present as landscapes the second round of clone screening results performed for the expression systems studied. Mean values for each and standard deviations are plotted and expressed in terms of activity units (AU) normalized per biomass concentration (OD_600_). S1A–P_*GAP*_-based clone GAP-C; S1B–P_*PDF*_-based clone PDF-C; S1C–P_*UPP*_-based clone UPP-C.

**Additional file 2: Table S2.A**, table listing the primer pairs used for gene dosage analyses and relative transcription levels (RTL) determination by means of ddPCR and qPCR, respectively. **B**, table that presents the gene dosage determination of CalB producer clones by digital droplet PCR (ddPCR). Analyses were performed by triplicates, using Actin gene (*ACT1*) as reference. Two positive controls which contain 3 and 5 copies of the expression cassette for *Candida rugosa* lipase 1 (*CRL1*) were also analyzed as controls.

**Additional file 3: Figure S3.** Figure that presents the analysis of the product quality by SDS-PAGE. Samples from fed-batch cultivations—PDF-C and UPP-C—run at A: µ_sp_ = 0.10 h^−1^ and B: µ_sp_ = 0.05 h^−1^ were analyzed. Different samples obtained at different feeding time (FT) supernatants were loaded on SDS-PAGE. BSA standards at different concentrations were also loaded in SDS-PAGE as reference (lanes 1–4). **A**. µ_sp_ = 0.10 h^−1^.PDF 7: 17h FT; PDF 8: 18h FT; PDF 9: 20h FT; PDF 10: 21h FT; UPP 8: 19h FT; UPP 9: 21h FT; UPP 10: 22h FT. **B**. µ_sp_ = 0.05 h^−1^. PDF 1: Batch end, PDF 5: 19h FT; PDF 7: 24h FT; PDF 8: 27h FT; PDF 9: 36h FT; PDF 10: 41h FT; PDF 11: 43h FT; UPP 1: Batch end, UPP 7: 27h FT; UPP 8: 37h FT; UPP 9: 40h FT; UPP 10: 42h FT; UPP 11:44h FT.

## Data Availability

The datasets and materials used and/or analysed during the current study are available from the corresponding author on reasonable request.
