# Supplementary material for: Bioprocess performance analysis of novel methanol-independent promoters for recombinant protein production with Pichia pastoris
Source: Microb Cell Fact. 2021 Mar 23;20:74. doi: 10.1186/s12934-021-01564-9 (PMC7986505; doi:10.1186/s12934-021-01564-9)

**Additional file 1: S1.** Figures that present as landscapes the second round of clone screening results performed for the expression systems studied. Mean values for each and standard deviations are plotted and expressed in terms of activity units (AU) normalized per biomass concentration (OD_600_). **S1A** – P*_GAP_*-based clone GAP-C; **S1B** – P*_PDF_*-based clone PDF-C; **S1C** – P*_UPP_*-based clone UPP-C.


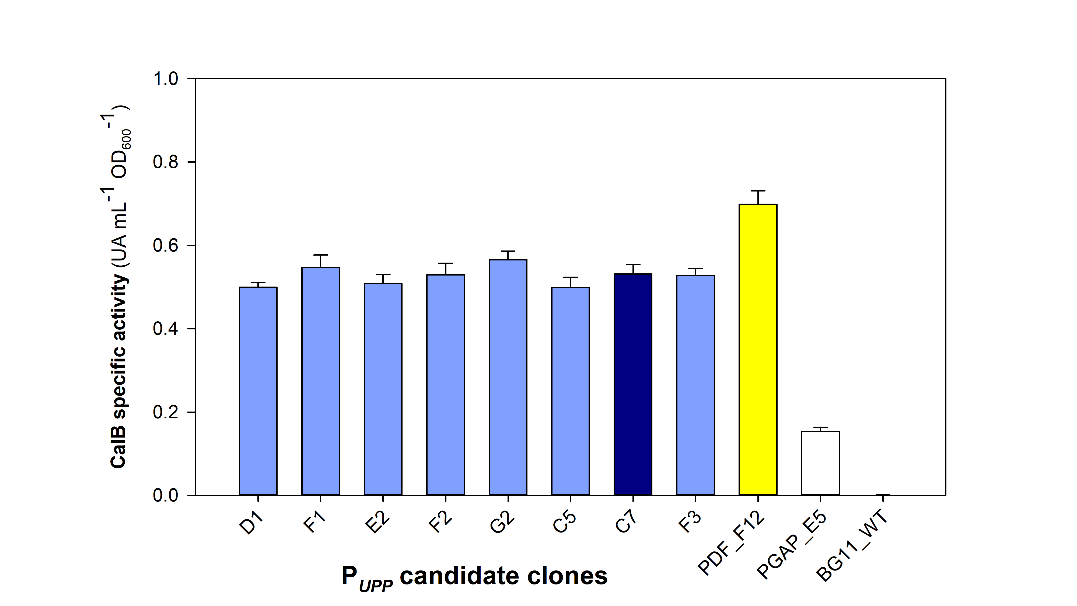

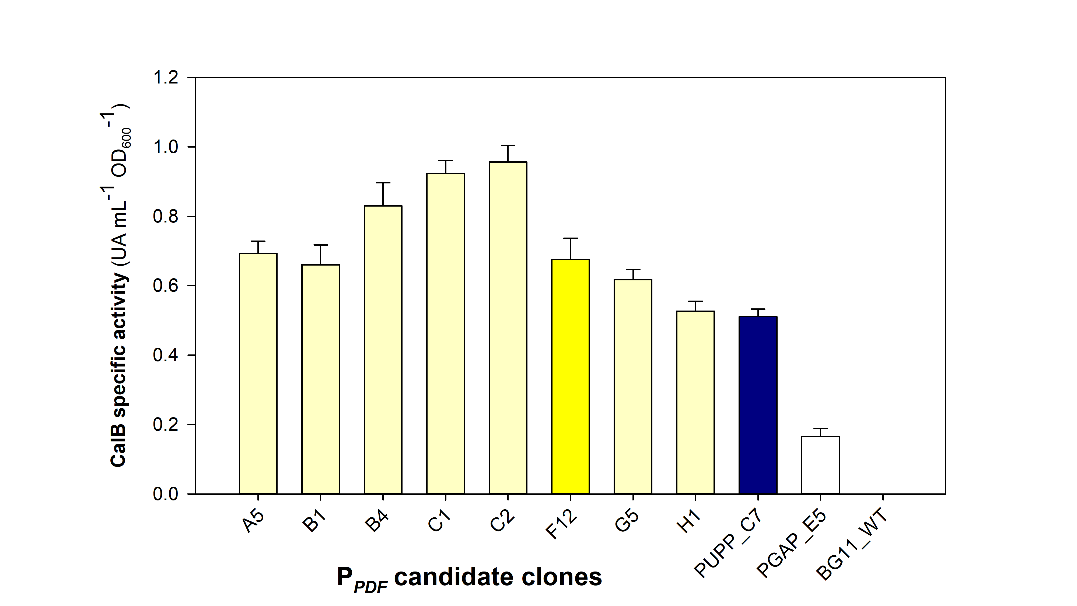

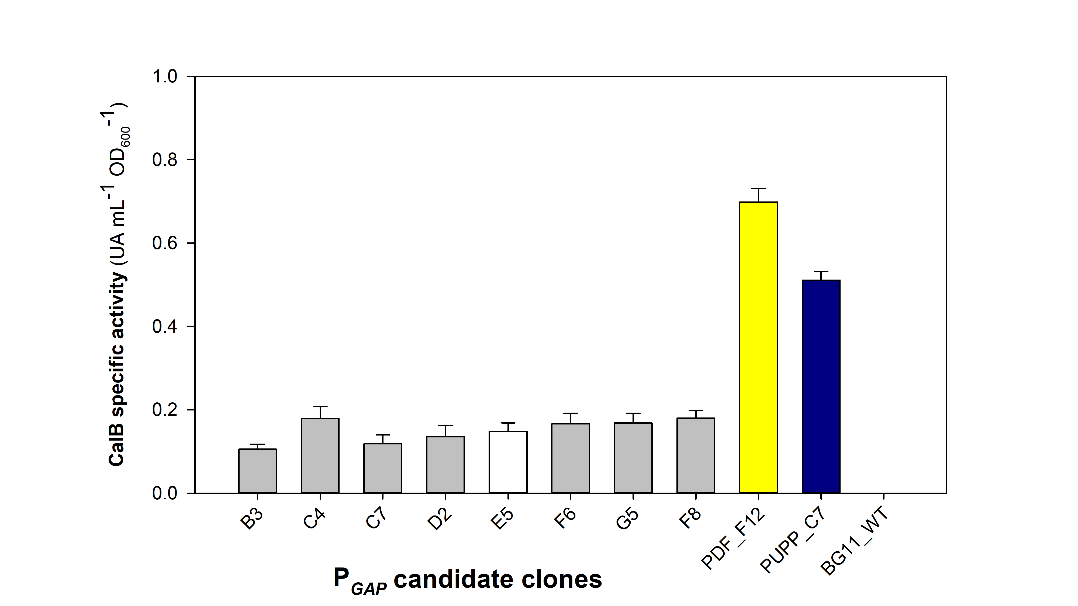

Supplement: Supplementary file 1 — Additional file 1: Table S1. Figures that present as landscapes the second round of clone screening results performed for the expression systems studied. Mean values for each and standard deviations are plotted and expressed in terms of activity units (AU) normalized per biomass concentration (OD600). S1A–PGAP-based clone GAP-C; S1B–PPDF-based clone PDF-C; S1C–PUPP-based clone UPP-C. [file 12934_2021_1564_MOESM1_ESM.docx]
