# Supplementary material for: Bioprocess performance analysis of novel methanol-independent promoters for recombinant protein production with Pichia pastoris
Source: Microb Cell Fact. 2021 Mar 23;20:74. doi: 10.1186/s12934-021-01564-9 (PMC7986505; doi:10.1186/s12934-021-01564-9)
Supplement: Supplementary file 2 — Additional file 2: Table S2.A, table listing the primer pairs used for gene dosage analyses and relative transcription levels (RTL) determination by means of ddPCR and qPCR, respectively. B, table that presents the gene dosage determination of CalB producer clones by digital droplet PCR (ddPCR). Analyses were performed by triplicates, using Actin gene (ACT1) as reference. Two positive controls which contain 3 and 5 copies of the expression cassette for Candida rugosa lipase 1 (CRL1) were also analyzed as controls. [file 12934_2021_1564_MOESM2_ESM.docx]

**Additional file 2: S2 A.** Primer pairs used for gene dosage analyses and relative transcription levels (RTL) determination by means of ddPCR and qPCR, respectively.

| **#** | **Name** | **Product** | **Function in analysis** | **Sequence** |
| --- | --- | --- | --- | --- |
| 1 | Act1_FW | ACT1 | ddPCR gene dosage housekeeping | CCTGAGGCTTTGTTCCACCCATCT |
| 2 | Act1_RE |  |  | GGAACATAGTAGTACCACCGGACATAACGA |
| 3 | MTH1_FW | MTH1 | qPCR Transcription analysis  housekeeping | TACGACATGGTTCCTCCCCCTTG |
| 4 | MTH1_RE |  |  | CGTTTCCTCTTGGACTCGTCTATCGTC |
| 5 | CALB_FW | CALB | qPCR Transcription analysis  Target protein | GATCAACCACTGGTCAGGCAAGATC |
| 6 | CALB _RE |  |  | CGCAATTCTGTTTAGGACCTGCAACG |
| 7 | KAR2_FW | KAR2 | qPCR Transcription analysis  UPR reporter | GGTAGTCATAACGCCACCAGTAGTCTC |
| 8 | KAR2_RE |  |  | GGAATTAACCCAGATGAAGCTGTCGC |
| 9 | HAC1_FW | HAC1 | qPCR Transcription analysis  UPR reporter | GGTTGGAAGCCTTAGGTGGTACCG |
| 10 | HAC1_RE |  |  | CCTCAGTCAAAGATCTGCGAGTGG |
| 11 | ERO1_FW | ERO1 | qPCR Transcription analysis  UPR reporter | GGAATGGTGATGAGGGATTCTGCAAG |
| 12 | ERO1_RE |  |  | GAGGACAGCTCATTTTCATCTTGGCCC |

**Additional file 2: S2 B.** Results of gene dosage determination of CalB producer clones by digital droplet PCR (ddPCR). Analyses were performed by triplicates, using Actin gene (*ACT1*) as reference. Two positive controls (PC) which contain 3 (PC1) and 5 (PC2) copies of the expression cassette for *Candida rugosa* lipase 1 (*CRL1*) were also analyzed as controls. R1: replicate 1; R2: replicate 2; R3: replicate 3; Avg: average; SD: standard deviation; GD: gene dosage.

|  | **Positive droplets** | | | | | | | | | |  |  |
| --- | --- | --- | --- | --- | --- | --- | --- | --- | --- | --- | --- | --- |
|  | ***ACT1*** | | | | | ***CALB*** | | | | |  |  |
| **Clone** | R1 | R2 | R3 | Avg | % SD | R1 | R2 | R3 | Avg | % SD |  | ***GD*** |
| **GAP-C** | 3165 | 3189 | 3025 | 3126 | 2,83 | 3016 | 3198 | 3078 | 3097 | 2,99 |  | **0,99** |
| **PDF-C** | 2536 | 2438 | 2491 | 2488 | 1,97 | 2849 | 2671 | 2712 | 2744 | 3,40 |  | **1,10** |
| **UPP-C** | 2849 | 2741 | 2856 | 2815 | 2,29 | 2901 | 2846 | 2873 | 2873 | 0,96 |  | **1,02** |

|  | **Positive droplets** | | | | | | | | | |  |  |
| --- | --- | --- | --- | --- | --- | --- | --- | --- | --- | --- | --- | --- |
|  | ***ACT1*** | | | | | ***CRL1*** | | | | |  |  |
| **PC** | R1 | R2 | R3 | Avg | % SD | R1 | R2 | R3 | Avg | % SD |  | ***GD*** |
| **PC1** | 4629 | 4519 | 4872 | 4673 | 3,87 | 14598 | 14493 | 14964 | 14685 | 1,68 |  | **3,14** |
| **PC2** | 3058 | 3214 | 3187 | 3153 | 2,64 | 15267 | 15036 | 16001 | 15435 | 3,26 |  | **4,90** |
