# Supplementary material for: Bioprocess performance analysis of novel methanol-independent promoters for recombinant protein production with Pichia pastoris
Source: Microb Cell Fact. 2021 Mar 23;20:74. doi: 10.1186/s12934-021-01564-9 (PMC7986505; doi:10.1186/s12934-021-01564-9)
Supplement: Supplementary file 3 — Additional file 3: Figure S3. Figure that presents the analysis of the product quality by SDS-PAGE. Samples from fed-batch cultivations—PDF-C and UPP-C—run at A: µsp = 0.10 h−1 and B: µsp = 0.05 h−1 were analyzed. Different samples obtained at different feeding time (FT) supernatants were loaded on SDS-PAGE. BSA standards at different concentrations were also loaded in SDS-PAGE as reference (lanes 1–4). A. µsp = 0.10 h−1.PDF 7: 17h FT; PDF 8: 18h FT; PDF 9: 20h FT; PDF 10: 21h FT; UPP 8: 19h FT; UPP 9: 21h FT; UPP 10: 22h FT. B. µsp = 0.05 h−1. PDF 1: Batch end, PDF 5: 19h FT; PDF 7: 24h FT; PDF 8: 27h FT; PDF 9: 36h FT; PDF 10: 41h FT; PDF 11: 43h FT; UPP 1: Batch end, UPP 7: 27h FT; UPP 8: 37h FT; UPP 9: 40h FT; UPP 10: 42h FT; UPP 11:44h FT. [file 12934_2021_1564_MOESM3_ESM.docx]

**Additional file 3: S3.**

Figure that presents the analysis of the product quality by SDS-PAGE. Samples from fed-batch cultivations —PDF-C and UPP-C— run at A: µ_sp_ = 0.10 h^-1^ and B: µ_sp_ = 0.05 h^-1^ were analyzed. Different samples obtained at different feeding time (FT) supernatants were loaded on SDS-PAGE. BSA standards at different concentrations were also loaded in SDS-PAGE as reference (lanes 1-4).

-
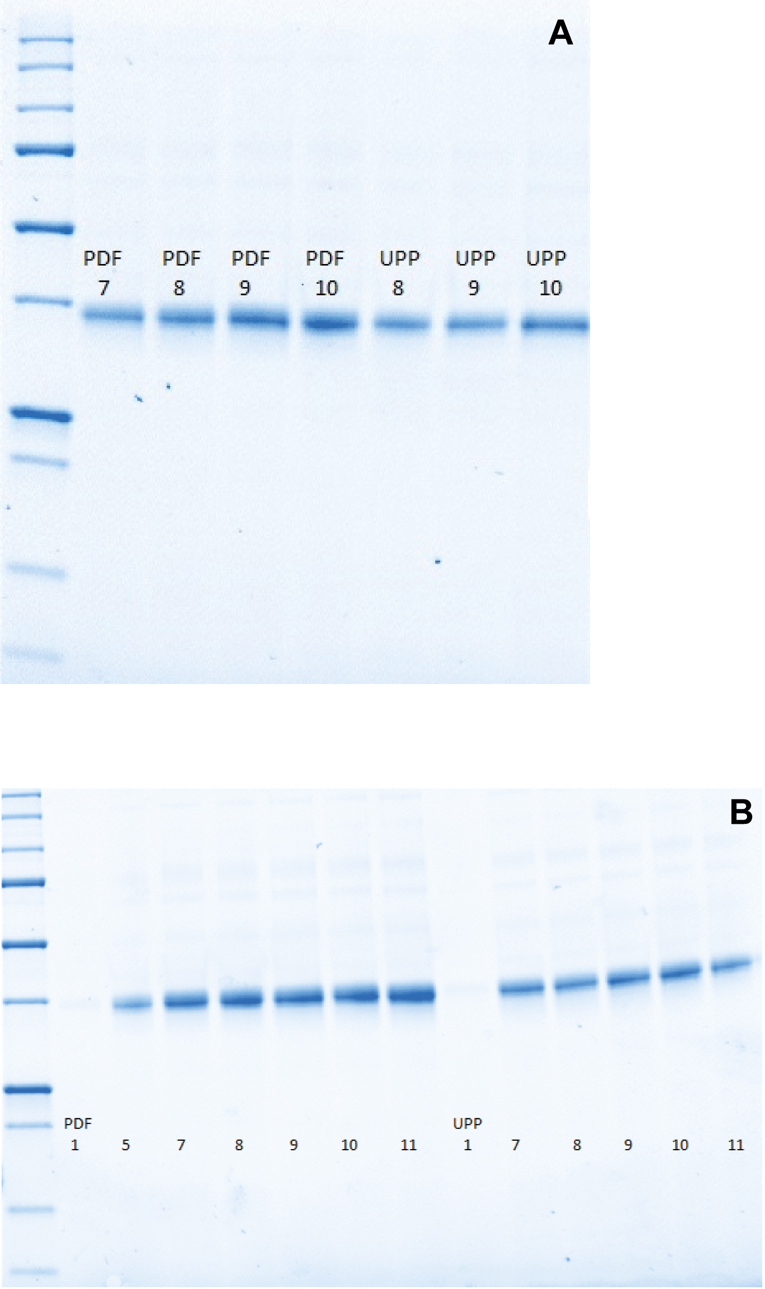
**A) µ_sp_ = 0.10 h^-1^**

**PDF 7:** 17h FT; **PDF 8:** 18h FT; **PDF 9:** 20h FT; **PDF 10:** 21h FT; **UPP 8:** 19h FT; **UPP 9:** 21h FT; **UPP 10:** 22h FT.

-
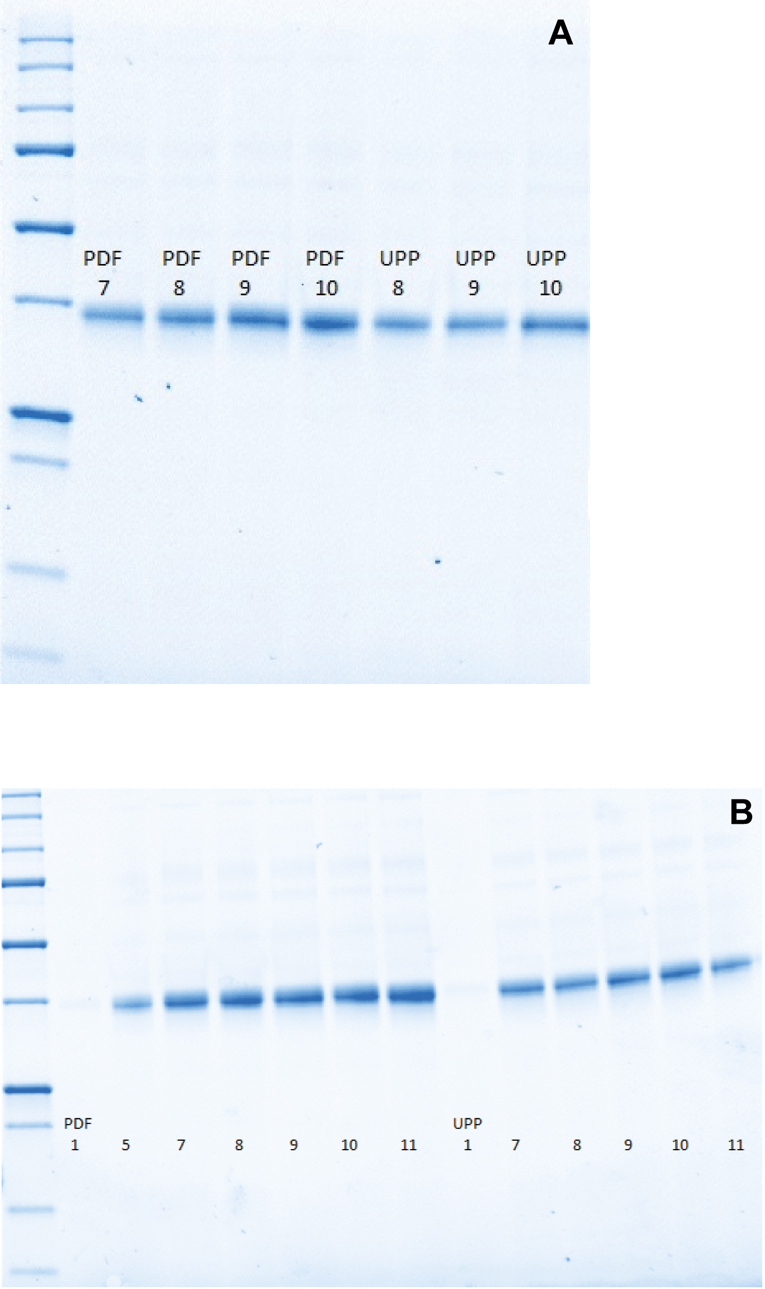
**B) µ_sp_ = 0.05 h^-1^**

**PDF 1:** Batch end, **PDF 5:** 19h FT; **PDF 7:** 24h FT; **PDF 8:** 27h FT; **PDF 9:** 36h FT; **PDF 10:** 41h FT; **PDF 11:** 43h FT; **UPP 1:** Batch end, **UPP 7:** 27h FT; **UPP 8:** 37h FT; **UPP 9:** 40h FT; **UPP 10:** 42h FT; **UPP 11:**44h FT.
